# Visual perspective and the characteristics of mind wandering

**DOI:** 10.3389/fpsyg.2013.00699

**Published:** 2013-10-09

**Authors:** Brittany M. Christian, Lynden K. Miles, Carolyn Parkinson, C. Neil Macrae

**Affiliations:** ^1^School of Psychology, University of AberdeenAberdeen, UK; ^2^Department of Psychological and Brain Sciences, Dartmouth CollegeHanover, NH, USA

**Keywords:** mental imagery, visual perspective, vantage point, mind wandering, cross cultural, third person

## Abstract

When the mind wanders away from the here-and-now toward imaginary events, it typically does so from one of two visual vantage points—a first-person perspective (i.e., the world is seen as it is in everyday life) or a third-person perspective (i.e., the world is seen from the viewpoint of an outside observer). While extant evidence has detailed consequences that ensue from the utilization of these distinct points of view, less is known about their more basic properties. Here, we investigated the prevalence, demographics and qualities associated with the visual perspective that people spontaneously adopt when the mind wanders. The results from a cross-cultural survey (*N* = 400) revealed that almost half of the participants (46%) typically utilize a third-person perspective when mind wandering. Further, culture and gender were shown to impact the distribution of first- and third-person imagers. Specifically, a first-person perspective was more common among participants from Western nations and females, while participants from Eastern cultures resonated more strongly with a third-person perspective. Moreover, these factors were also shown to impact qualities (e.g., temporal locus, vividness) of mental imagery. Taken together, the current findings elucidate the prevalence of first- and third-person visual perspectives and detail individual differences that influence the qualia of mind wandering.

## Introduction

Despite being physically grounded in the present, people spend up to half of their waking lives mentally detached from the here-and-now (Giambra, 1995; Smallwood et al., 2003, 2004; Schooler et al., 2005; Smallwood and Schooler, 2006; Kane et al., 2007; Killingsworth and Gilbert, 2010). In particular, it is when plagued by foul moods (Smallwood et al., 2009) or performing repetitive or mundane activities (Fisher, 1987; Singer, 1966) that the mind is most likely to wander. This transcendence of time and space reorients attention inward, to a world of vibrant mental images that are often unrelated to the task at hand.

Given the mind's propensity to escape lackluster or troublesome realities, one cannot help but speculate about the imaginative characters and properties these fantasy destinations contain. Perhaps disappointingly, it is rarely lions, witches and magic wardrobes, but rather house cats, horrible bosses and overstuffed closets that are typically encountered when meandering through the mental world. In short, the contents of the wandering mind most commonly reflect the typical characteristics of day-to-day life (Addis et al., 2007; Buckner and Carroll, 2007; Schacter et al., 2007; Szpunar, 2010). This overlap between real and imaginary events reveals the building blocks used to construct the mental world. Specifically, it is only by recycling episodic and semantic knowledge that it is possible to simulate future realities at all (Szpunar, 2010).

While at first glance mental images that emphasize the mundane aspects of life may seem futile, they are actually an extremely prudent use of the mind's power of simulation—facilitating matchless preparation for things to come. As is evidenced by the temporal locus of mental journeys, people do not tune out simply to watch reruns of the past, but more often to problem solve in the present and gain sneak previews of the future (Binder et al., 1999; Klinger, 1999; Mason et al., 2009; Killingsworth and Gilbert, 2010). Consequently, the time spent mind wandering can often provide functional advantages. For example, mentally simulating a route home from work on a Friday afternoon or the flavor of a bacon-infused cocktail can help one avoid traffic jams or circumvent a potentially disastrous beverage choice. To this end, mind wandering comprises a core component of mental functioning, facilitating decision making and action planning (Tulving, 2002; Schacter et al., 2007).

It is precisely because thinking serves as a functional precursor of doing (James, 1890; Fiske, 1992) that the world tends to be imagined as it is experienced in everyday life, preserving its structural properties and activating much the same neural architecture that supports genuine experience (Jeannerod, 2001; Fadiga and Craighero, 2004; Ganis et al., 2004; Kosslyn et al., 2006). However, one notable inconsistency between the real and the imagined persists. During mental simulations, two visual perspectives are possible: a first-person (actor) perspective and a third-person (observer) perspective. With a first-person visual experience, the world is imagined just as it is encountered in everyday life, viewing only what would actually lie within one's own visual field. Alternatively a third-person visual perspective mimics the viewpoint of an outsider^1^, allowing people to visualize themselves in their mind's eye and observe their own behaviors (Nigro and Neisser, 1983; Sutin and Robins, 2008). So while real life only occasionally affords the opportunity to view oneself from a third-person perspective (e.g., photographs, mirrors), the imagination can readily recruit this point of view (Nigro and Neisser, 1983; Sutin and Robins, 2008; Rice and Rubin, 2011).

Of particular relevance to the manner in which imagination can shape behavior, a growing body of evidence suggests that multiple vantage points are not simply an innocuous aspect of conscious experience, but rather exert influence on the consequences of mental simulation (White and Hardy, 1995; Janzen et al., 2001; Libby et al., 2007; Macrae et al., 2013). These observed differences have often been attributed to the type of information that is most readily accessed from a given perspective (Libby and Eibach, 2011). More specifically, first-person perspective has been shown to emphasize experiential aspects of a simulation whereas a third-person perspective seems to incorporate more contextual information. By providing access to unique types of information, visual perspective can alter the emotional intensity of an event (Berntsen and Rubin, 2006; Williams and Moulds, 2008) and influence cognitive processing styles (Libby and Eibach, 2011).

While perspective can be overtly manipulated, influencing everything from the way an event is construed (Libby and Eibach, 2011) to the extent that an imaginary experience will influence subsequent judgments (e.g., person perception; Macrae et al., 2013) and behaviors (e.g., voting; Libby et al., 2007), the wandering mind will spontaneously adopt one of the two visual perspectives when playing stimulus independent thoughts in the theater of consciousness. As such, might the perspective naturally utilized for simulation also be indicative of the types of information emphasized when the mind wanders? Despite notable influences on cognition and behavior when perspective is manipulated in a laboratory, little is known about the characteristic properties of the mental worlds that people spontaneously construct. Instead, the majority of research on perspective taking has utilized memory-probe paradigms (Sutin and Robins, 2008). As a result, it is uncertain just how common a third-person perspective of self is in the mental world and whether or not there are systematic patterns with respect to the visual perspective individuals most frequently adopt when their minds wander. Put simply, it is still unclear *which* visual perspective is most prevalently adopted and by *whom*.

When Nigro and Neisser (1983) originally identified that first- and third-person perspectives were utilized in recall, they reported a greater overall occurrence of field (i.e., first-person) than observer (i.e., third-person) memories. While the actual proportion of memories characterized by a third compared to first-person visual perspective may be greater than initially thought (Rice and Rubin, 2011), there seems to be biases toward a given perspective (e.g., a greater amount of memories characterized by either first or third point of view) between participants (Nigro and Neisser, 1983). As a result of this finding, Nigro and Neisser (1983) speculated that a commonly utilized point of view might be indicative of different cognitive processing styles across participants (Nigro and Neisser, 1983).

Evidence corroborating the conjecture that individual differences may influence point of view has suggested that conceptualizations of the self and its relation to the environment are linked to the adoption of a spatial-visual perspective (Cohen et al., 2007; Libby and Eibach, 2011). Cohen et al. (2007) have demonstrated that Asian-Americans and Euro-Americans show systematic differences in point of view depending upon the context of a recalled memory. Specifically, when recalling memories that focus on the self, Asian-Americans are more likely to adopt the perspective of an outside observer than are Euro-Americans. These patterns of adopted visual perspective are believed to be reflective of distinct socio-cultural processing styles. Indeed, a cultural divide in conceptualizations of self such that Easterners are said to be more collectivistic, whereas Westerners tend to be more individualistic, (for reviews, see Triandis, 1989; Oyserman et al., 2002) provides a cogent explanation for the patterns observed in perspectives utilized in personal memories. As such, it seems reasonable to suspect that deeply engrained cognitive processes will be manifest globally during mind wandering, leading people to resonate most strongly with a particular viewpoint.

Following this line of reasoning, we suspect that when asked to characterize the visual perspective most frequently adopted during mind wandering: (i) both first- and third-person points of view will be commonly identified as dominant modes of simulation (across individuals); and (ii) vantage point preferences are likely to vary as a function of factors associated with population-level differences in processing style (e.g., culture). Specific predictions, based on previous work by Cohen et al. (2007) and an in-depth body of knowledge explicating cultural differences in processing style (i.e., holistic vs. analytic, see Barrett et al., 1998; Seidlitz and Diener, 1998; Nisbett and Masuda, 2003; Piefke et al., 2005) and conceptualizations of self (i.e., individualistic vs. collectivistic), focus on the influence of culture on visual perspective. Due to the self-centric nature of mind wandering (Baird et al., 2011), we anticipate cultural differences to manifest themselves in dominant imagery perspectives. Specifically, we suspect participants from Eastern societies will resonate more strongly with a third-person perspective than participants from Western Societies (i.e., similar to the patterns observed by Cohen et al., 2007). While the current investigation also explored demographic variables such as age and gender that have been shown to influence visual perspective during memory retrieval (Huebner and Fredrickson, 1999) and day dreaming (Mar et al., 2012) we remained agnostic in regards to what effect these variables might have on the visual perspective participants use to characterize their mind wandering.

It is important to note the current paper is exploratory and descriptive in nature, and was initially motivated to explore the extent to which participants most commonly resonated with a first-or third-person perspective outside of a laboratory setting. While previous work has identified the ability and tendency for people to utilize both first- and third-person perspectives (even within a single memory, Rice and Rubin, 2009) the methods we employed isolated what individuals' believe to be their most common or dominant visual perspective. The research was inspired by observations in the laboratory when instructing participants to take either a first- or third-person perspective. Namely, a number of individuals reported struggling to maintain a visual perspective that was “unfamiliar to them” or counter to what they “normally do.” While the ability for participants to tap into and accurately assess the nature of their visual imagery could be questioned, it seems more likely that these reports are indicative of a phenomenon that has been largely overlooked in the perspective taking literature since it was initially suggested by Nigro and Neisser (1983)—people may have a natural or dominant visual perspective.

The current study recruited an international sample to investigate the prevalence of first- and third-person natural imagery perspectives as well as the influence of demographic variables (culture, gender, age) on the view from the mind's eye. Additionally, we considered the effects that these factors may have on other core properties (e.g., vividness, temporal locus, valence, sensory modality) of the mental events that occur when the mind wanders as these variables are commonly assessed when considering mind wandering, day dreaming and other related topics.

## Materials and methods

### Ethics statement

The study was reviewed and approved by the School of Psychology, University of Aberdeen Ethics Committee. All participants were informed about the nature of the study prior to agreeing to participate and were able to withdraw from the study at any point.

### Participants

Four hundred participants (197 females) aged 16–63 years^2^ (*M* = 29.4 years, *SD* = 10.0 years) were recruited and tested online using Amazon's Mechanical Turk^3^ (www.mturk.com). Two hundred of the participants tested were residing in Eastern Asian Countries (e.g., India, South Korea) while the remaining two hundred were residing in Western Countries (e.g., U.S., U.K.)^4^. Mechanical Turk's unique “worker id” numbers were recorded for each participant, thus allowing multiple responses from the same individual to be excluded from data analysis.

### Materials and procedure

A short questionnaire was designed to investigate fundamental characteristics of spontaneous mind wandering. After reporting on basic demographic information (age, sex, country of residence), participants were asked to identify the visual perspective (i.e., first or third) they most commonly adopt when their mind wanders or they imagine an event by selecting one of the following options: 1. “I cannot see myself (I imagine the scene through my own eyes)” or 2. “I can see myself (I imagine the scene as if I were an outside observer).” Next participants reported, on a 10-point scale, the typical vividness (1 = not very vivid, 10 = very vivid), valence (1 = always negative, 10 = always positive), and temporal direction (1 = always past, 10 = always future) of their mental meanderings. Finally, participants were asked to identify the primary sensory modality (e.g., vision, audition) experienced during instances of mental imagery.

## Results

### Frequency of perspective

The primary focus of the present study was to identify the frequency with which first- and third-person visual perspectives are spontaneously adopted during periods of mind wandering and mental imagery. To this end, 53% of participants reported most commonly utilizing a first-person visual perspective while the remaining 47% reported adopting a third-person vantage point when imagining events^5^. A chi-square test of goodness-of-fit (expected values = 50%) confirmed that these proportions were uniformly distributed, χ^2^(1,*N* = 400) = 1.44, *p* = 0.23, such that, overall, both visual perspectives were equally prevalent. Subsequent analyses sought to further explore this finding by identifying if the distribution of first and third visual perspectives differed as a function of demographic variables such as age, gender and culture.

### Culture

**Figure 1:**
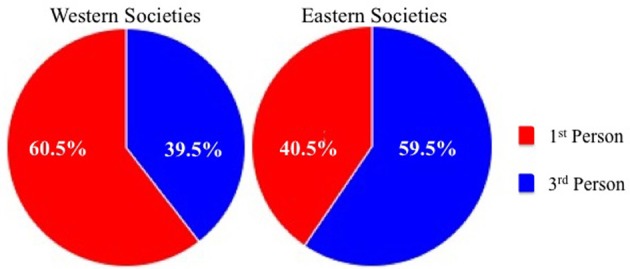
**Percentage of reported visual perspective in Western (left panel) and Eastern (right panel) societies**.

A chi-square test of independence revealed a relationship between culture (i.e., West vs. East) and visual perspective, χ^2^(1,N = 400) = 9.03, *p* = 0.003. Specifically, in Western cultures a first-person perspective was most prevalent (60.5%), while in Eastern cultures this pattern was reversed such that a third-person perspective was most frequently reported (59.5%), as shown in Figure 1. Subsequent chi-square tests of goodness-of-fit conducted separately for each culture confirmed a preference for first-person imagery among those from Western cultures, χ^2^(1,*N* = 200) = 8.82, *p* = 0.003, and third-person imagery within those from Eastern cultures, χ^2^(1,*N* = 200) = 7.22, *p* = 0.007.

### Gender

**Figure 2:**
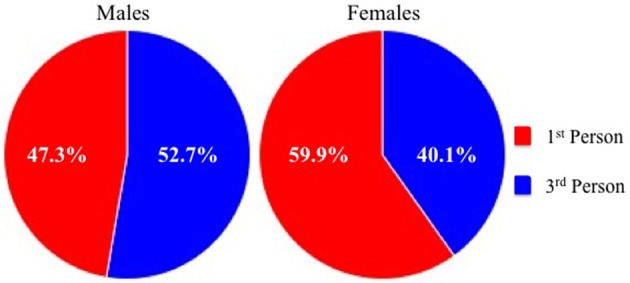
**Percentage of reported visual perspective among male (left panel) and female (right panel) participants**.

A chi-square test of independence revealed a relationship between gender and visual perspective, χ^2^(1,*N* = 400) = 5.39, *p* = 0.02. Female participants were more likely to report adopting a first-person perspective (59.9%) than their male counterparts (47.3%), as shown in Figure 2. Furthermore, separate chi-square tests of goodness-of-fit revealed that while females showed a preference (greater than chance) for first-person over third-person imagery, χ^2^(1,*N* = 197) = 6.22, *p* = 0.01, males showed no such pattern, χ^2^(1,*N* = 203) = 0.60, *p* = 0.44.

### Age

A point-biserial correlation revealed no relationship between participant age and the visual perspective they reported adopting during mental imagery, *r*_*pb*_(400) = 0.008, *p* = 0.88.

### Characteristics of imagery

Separate 2 (Culture: West or East) × 2 (Gender: Male or Female) between-participants ANOVAs were conducted on participants' ratings of the typical vividness, valence and temporal direction of their imaginary experiences.

#### Vividness

A main effect of Culture, *F*_(1, 396)_ = 5.33, *p* = 0.02, η_*p*_^2^ = 0.01, was revealed such that participants from Eastern societies reported less vivid mental imagery (*M* = 5.58, *SD* = 2.50) than participants from Western societies (*M* = 6.17, *SD* = 2.09). Furthermore, a main effect of Gender, *F*_(1, 396)_ = 5.38, *p* =0.02, η_p_^2^ = 0.01, was also revealed whereby males reported less vivid mental imagery (*M* = 5.58, *SD* = 2.30) than females (*M* = 6.18, *SD* = 2.30). No interaction effect was found.

#### Valence

Participants' reports of the valence of their mental images did not vary as a function of Culture or Gender, nor was there any interaction between these factors.

#### Temporal direction

A main effect of culture on the temporal direction of reported mental imagery was revealed, *F*_(1, 396) = 12.38_, *p* < 0.001, η_*p*_^2^ = 0.03, whereby participants from Eastern cultures reported a greater tendency toward future-oriented mental imagery (*M* = 5.85, *SD* = 2.57) than participants from Western cultures (*M* = 5.04, *SD* = 2.24). No other main effects or interaction effects were found for ratings of temporal direction.

#### Sensory modality

Finally, irrespective of culture and gender, vision and audition were reported as the most dominant sensory modalities of mental imagery. In total, 82% (328) of participants reported vision as the primary sensory component of their mental imagery. Interestingly, 7.8% (31) reported their primary sensory modality to be audition, 3.8% (15) tactile, 3.3% (13) kinesthetic, 1.8% (7) olfaction, and 1.5% (6) gustatory.

## Discussion

The current study explored whether the core properties of the mental imagery that is often associated with mind wandering vary as a function of participants' demographic profile. Most notably, this investigation revealed that approximately half (46%) of participants reported most commonly adopting a third-person visual perspective when their minds wandered or they imagined an event. Interestingly, further analysis revealed that visual perspective differed as a function of demographic variables such that females and residents from Western nations reported most frequently adopting a first-person point of view, whereas a third-person perspective was more common among participants from Eastern countries.

The overall dominance of first-person visual perspective in Western cultures is consistent with findings from memory-probe experiments (Nigro and Neisser, 1983) and other investigations of spontaneously adopted visual perspective during guided mental imagery (Robinson and Swanson, 1993). To our knowledge, the prevalence of first- and third-person visual perspectives has not been studied exclusively in Eastern Societies, although Asian-Americans have been shown to utilize a third-person perspective during memory retrieval more readily than Euro-Americans (Cohen et al., 2007). The current findings confirm and extend previous work, showing that patterns of visual perspective differ across cultures and emphasize the prevalence of third-person simulations in everyday life (Rice and Rubin, 2011).

Focusing specifically on gender, it should be noted that a few reports have shown females to utilize a third-person perspective more than males (Huebner and Fredrickson, 1999; Rice and Rubin, 2009). A number of factors may be responsible for the differences between these findings and the ones presented in the current paper. Namely, previous experiments have focused on memory and dictated specific content, which is likely to influence the perspective adopted during simulation (Rice and Rubin, 2009). For example, Rice and Rubin (2009) found females to have more dominant third-person experiences than males when recalling memories prior to the first grade. Additionally, asked to recall memories that were likely to makes individuals feel objectified, females were more likely than males to utilize a third-person as opposed to a first-person perspective (Huebner and Fredrickson, 1999). It is precisely because manipulations within laboratory studies can so readily shape the visual perspective a person adopts that we chose to address the prevalence of first- and third-person viewpoints with a survey approach. However, establishing whether dissimilarities in methods can account for differences in findings (e.g., with respect to gender) needs to be addressed in future research.

Beyond vantage point, the present work identified other components of mental imagery that were also influenced by demographic variables. Specifically, culture and gender displayed systematic relationships with vividness ratings such that males and Easterners reported less vivid imagery than females and Westerners. Temporal locus (i.e., past vs. future) was also influenced by geographical location as individuals from Eastern societies reported a higher prevalence of future-oriented imagery than their Western counterparts. Characteristics such as valence and primary sensory modality, on the other hand, were unaffected by demographic variables.

Different imagery experiences across cultures and genders lead one to consider what factors might be shaping the characteristics of mind wandering. Previous work has postulated that differences in cognitive processing styles may underlie the tendency to adopt a given perspective during memory retrieval (Nigro and Neisser, 1983; Cohen et al., 2007). In other words, it might be the case that relatively stable differences in the way one conceptualizes the world also shape the visual perspective used to mentally simulate events. Indeed, research has indicated that an interaction between conceptualizations of self (i.e., independent vs. interdependent) and imagery content are associated with systematic shifts in perspectives between Asian-Americans and European-Americans during memory retrieval and online experiences (Cohen et al., 2007).

What is more, research on visual perspective has emphasized the role of first-person perspective in emotional/experiential simulations. While not tested here, it is possible that group level differences in the emotional intensity of mind wandering content (whether positive or negative) may influence natural imagery perspective and characteristics of imagery such as vividness. Indeed, previous research has identified that females (Davis, 1999) and Westerners (Wang, 2001) report more detailed and emotionally charged childhood memories. As emotionally intense memories are highly correlated with vividness (Talarico et al., 2004), these differences in episodic content may not only influence prospective future thought, but the assessments of general mind wandering characteristics. An important area for future research will be to investigate the extent to which these factors are associated with natural imagery perspective.

Since the current results speak to unconstrained mind wandering (i.e., mental content was not dictated, prompted, or probed), it is possible that the culture and gender differences seen here are a result of reliable differences in *what* is most commonly simulated (i.e., imagery content), *how* it is contextualized (i.e., cognitive processing style) or a combination of the two. Future research could investigate typical content of mind wandering as it relates to demographic variables such as gender and culture. If, for instance, residents of Eastern societies are more likely to simulate social situations (i.e., emphasizing relationship with group) or females are more likely to imagine intensely emotional scenarios (i.e., emphasizing internal experience), then these differences may underlie the unique recruitment of first vs. third-person visual perspectives.

Elsewhere, it has been shown that specific types of information seem to be most commonly associated with each point of view. Namely, a first-person perspective has been shown to provide greater access to the internal/emotional components (experiential factors) of a mental event whereas a third-person perspective is more likely to emphasize the contextual information about an imaginary experience (see Libby and Eibach, 2011). While speculative, it may be the case that first- and third-person perspectives bring different aspects of an experience into focus which, in turn, interacts with societal norms across cultures and genders (i.e., to express vs. suppress emotion, to understand self in relation to the group vs. an individual). As a result, certain types of simulations may recruit specific points of view. Further explorations could look at the relationship between natural imagery perspective and how individuals vary in terms of emotional expressivity or independence/interdependence as well as other individual difference measures.

Notwithstanding the necessity of future research to determine the additional factors that impact population level patterns in vantage point preference, a growing body of evidence testifies to the real world consequences that can transpire as a result of adopting a specific visual perspective. For example, the way that imaginary experiences shape core aspects of how we perceive others (Macrae et al., 2013), the likelihood of making a trip to the polls (Libby et al., 2007), assessments of self-change over time (Libby and Eibach, 2002) and the intensity of a traumatic memory (Williams and Moulds, 2008) can all be matters of visual perspective. If it is assumed that disparate simulation modes result in unique behavioral outcomes, and there is an established division between dominant imagery perspectives, an important area for future research will be to establish if there are fundamental differences in the behaviors of natural first- and third-person imagers.

Importantly, however, it is worth noting that a natural or preferred imagery perspective (as identified here) does not suggest that an individual would visualize every simulation through the lens of a single perspective, nor that they would be unable or unlikely to alternate between vantage points (Robinson and Swanson, 1993). Rather, we emphasize that people differentially resonate with each of the two possible visual perspectives, an idea that has been echoed in investigations of motor imagery (see Hall, 1997; Calmels et al., 2006; Holmes, 2007) and memory (Nigro and Neisser, 1983). As was previously noted, it is possible that the current results reflect a specific mental imagery simulation (i.e., “a simulation of simulations”) that was run and utilized to assess their personal imagery experiences. As such, the availability and type of content simulated may be shaping participants' responses to the question of a dominant imagery perspective. While we are confident that the present approach accurately reflects the way participants engage in the mental world^3^, it is of course, feasible that additional, unknown factors contributed to the current findings. For instance, if participants were in fact unable to accurately report about a dominant visual perspective, then a potentially interesting question would be to investigate the social and cultural factors that ultimately influenced their decisions in regards to what perspective they think is dominant. To address other potential explanations, it is important that future research attempt to substantiate the claims made here with alternative methods of assessing natural imagery perspective.

Anecdotally, participants have been known to report difficulties maintaining a visual perspective incongruent to the one they typically adopt—an effect that seems to interact with the nature of the imagined event. Thus, when instructing a specific visual perspective, novelty, the possible increase of cognitive load required to maintain an unfamiliar perspective, or specific demands of a given imaginary event (e.g., simulating an internal sensation such as pain) may also influence outcomes. In short, participants who are requested to use an atypical visual perspective may show different results than those asked to adopt a perspective consistent with their preferred point of view for the given situation. While previous evidence seems to support this conjecture, further investigations will be vital in order to understand the nuanced interactions between natural imagery mode and instructed perspective (Williams and Moulds, 2008; Callow and Roberts, 2010). As such, it might also important for future work to consider each individual's common mode of simulation when exploring the outcomes of both spontaneous and guided mental imagery.

One final cautionary consideration brought to light by the current study is the semantic overlap that exists in the literature on perspective taking. While the descriptions of third-person perspective in our study detailed self as both actor and observer, a majority of the literature about third-person perspective hinges upon a self-other distinction where self is the observer and an “other” is the observed (e.g., Vogeley and Fink, 2003; Jackson et al., 2006; Ames et al., 2008). Studies operating with these definitions assess key aspects of social cognition (e.g., empathy, mentalizing) and highlight interesting differences between self and other imagery, however they are potentially tangential to the everyday self-simulations that serve to tailor individual behavior. To this end, recent research has focused on instructing a third-person-self simulation mode where self is both the observer and the observed (Libby and Eibach, 2011; Macrae et al., 2013). Given the potential for confusion, it seems particularly relevant for future research to emphasize *who* the third-person is during a simulation^1^. As such, further explorations will be vital to establish the similarities and differences between third-person-other and third-person-self visuo-spatial tasks.

Taken together, the current findings highlight that a third-person perspective is not constrained to considerations of an “other” person as it is often conceptualized in the literature, nor is it simply an unconventional device that can be pulled out of the cognitive toolbox to alter thinking and behavior. Rather, a third-person perspective that focuses on self appears to be a stable and common mode of mental simulation. It seems that the proportion of third- as compared to first-person memories is higher than had initially been postulated (Rice and Rubin, 2011), in that almost 50% of the individuals tested here resonated most strongly with a third-person perspective. Given that both first- and third-self perspectives regularly operate during unconstrained mind wandering, this suggests that each can be functional in everyday life. Future research will be needed to elucidate if the consequences of mental simulations are influenced not only by the content of the mental world, but also by how we naturally view it.

### Conflict of interest statement

The authors declare that the research was conducted in the absence of any commercial or financial relationships that could be construed as a potential conflict of interest.

## Footnotes

^1^An important conceptual distinction concerns “who” precisely is doing the observing and “who” is the observed when mentally simulating events from a third-person perspective. Here we operationalized third person simulations to mean that the self was both the observer and observed.

^2^Distribution of age groups in the current sample: 16–17 = 0.5%, 18–35 = 79.75%, 36–65 = 16.25% and 66–73 = 3.5%.

^3^See Duran et al. (2011) concerning validity of data collection via Mechanical Turk.

^4^All participants classified as “Eastern” either reported residing in one of the following countries (Bangladesh, India, Indonesia, Pakistan, Saudi Arabia, Singapore, South Korea, Philippines) or in “Asia” generally. All participants classified as “Western” reported residing in one of the following countries (Australia, Canada, France, Germany, Greece, Ireland, Italy, Netherlands, New Zealand, Portugal, Sweden, UK, USA).

^5^In response to a reviewer's concern regarding the validity of this critical measure (i.e., whether participants are able to reliably identify, via introspection, the visual perspective they most commonly adopt) we surveyed 12 additional participants (6 females, mean age = 19.4 years, all residing in a Western nation). Specifically, we asked them to engage in a series of guided mental imagery tasks involving performing simple everyday actions (e.g., picking up an object) and to report the perspective they adopted on each occasion. We also asked them to indicate the perspective they most commonly adopt using the same question as in the main study (task order was counterbalanced across participants). Consistent with the main findings, 6 participants reported most commonly using a first-person perspective, and 6 a third-person perspective. Importantly, those who reported most commonly adopting a first-person perspective also spontaneously adopted this perspective more frequently during guided imagery than those who reported naturally adopting a third-person perspective, *t*_(9. 51)_ = 2.59, *p* = 0.03, *d* = 1.49. This suggests some degree of consistency between the visual perspective people report adopting when asked to reflect on their mental experiences, and that employed when actually engaging in imagery.
